# Temporary adaptations to sexual behaviour during the mpox outbreak in 23 countries in Europe and the Americas: findings from a retrospective cross-sectional online survey

**DOI:** 10.1016/S1473-3099(24)00531-0

**Published:** 2024-12

**Authors:** Mateo Prochazka, Pietro Vinti, Ana Hoxha, Andy Seale, Antons Mozalevskis, Rosamund Lewis, Ruben Mayorga Sagastume, Martha Scherzer, Leilia Dore, Meg Doherty

**Affiliations:** a Global HIV, Hepatitis and Sexually Transmitted Infections Programmes, World Health Organization, Geneva, Switzerland; b Health Emergencies Programme, World Health Organization, Geneva, Switzerland; c Joint Infectious Diseases Unit, World Health Organization Regional Office for Europe, Copenhagen, Denmark; d Behavioural and Cultural Insights Unit, World Health Organization Regional Office for Europe, Copenhagen, Denmark; e HIV, Hepatitis, Tuberculosis, and Sexually Transmitted Infections Unit, Pan American Health Organization, Washington, DC, USA

## Abstract

**Background:**

After rapid epidemic growth between May and August, 2022, new mpox diagnoses declined in Europe and the Americas, with low-level transmission continuing thereafter. Characterising the extent of behavioural adaptation, mpox vaccination, and mpox prevalence across these regions could improve our understanding of the transmission dynamics of the virus. We aimed to characterise the presence and duration of adaptations to sexual behaviour related to the emergence of mpox during the first year of the outbreak among affected communities in Europe and the Americas.

**Methods:**

This retrospective, cross-sectional online survey was conducted in 23 countries in Europe and the Americas between May 19 and May 31, 2023. The survey was advertised via four geospatial dating apps used by affected communities. Eligible participants were aged 18 years or older and identified as a gay man, a bisexual man, a man who has sex with men, as transgender, or as non-binary. We described and regionally compared the mpox prevalence, mpox vaccination rates (one dose or two doses of modified vaccinia virus Bavarian Nordic), and the extent and duration of behavioural adaptation during the outbreak. For these behavioural outcomes, we used regression analyses to estimate crude prevalence ratios (PRs) and adjusted prevalence ratios (aPRs) with 95% CIs.

**Findings:**

Of 17 428 individuals who completed the survey, 16 875 (96·8%) met the eligibility criteria and were included in the study. 1086 (6·4%) participants reported having mpox during the outbreak. Vaccination with at least one dose was reported by 4987 (29·6%) participants; 3502 (20·8%) reported two doses. Vaccination rates in Latin America and eastern Europe and the western Balkans were significantly lower than in western Europe and northern America (p<0·0001). Adaptations to sexual behaviour were reported by 8583 (50·9%) of 16 875 participants and across all regions; 3045 (35·5%) of these 8583 participants said they continued adapting their sexual behaviour until May, 2023. Participants who reported concerns about mpox (9884 [58·6%] of 16 875) were more likely to adapt their behaviour than those who did not report concerns (PR 2·43 [95% CI 2·34–2·53]). In adjusted regression models, participants who reported vaccination (aPR 0·25 [95% CI 0·21–0·28] for two doses and 0·43 [0·37–0·51] for one dose) or having had mpox (0·37 [0·30–0·44]) were less likely to continue adaptations than those who did not. Participants in Latin America or northern America were significantly more likely to adapt their sexual behaviour and to continue with adaptations than those in western Europe.

**Interpretation:**

Adaptations to sexual behaviour due to mpox were widespread, dynamic, and responded to evolving individual risk perceptions. We propose that the decline in mpox transmission seen at the end of 2022 resulted primarily from a combination of behavioural adaptation and naturally acquired immunity. As mpox vaccination is an important preventive measure, stark vaccine inequity highlights the need to increase access to mpox vaccines.

**Funding:**

WHO Contingency Fund for Emergencies.

## Introduction

Mpox is an infectious disease caused by the monkeypox virus (MPXV), which can be transmitted from person to person via direct contact, including sexual contact.^1^, ^2^ Since May, 2022, a global outbreak of mpox has affected 121 countries, with 102 997 laboratory-confirmed diagnoses worldwide and 223 deaths reported to WHO as of July 31, 2024.^3^, ^4^ Although countries in west and central Africa had reported increasing transmission over many years and notably since 2012, MPXV clade IIb rapidly expanded to Europe and the Americas in 2022, and subsequently to Asia throughout 2023.^3^

**Research in context**

**Evidence before this study**

The global spread of monkeypox virus (MPXV) clade IIb was first identified in May, 2022 among communities of gay men, other men who have sex with men, transgender and gender diverse people, and sex workers. A rapid decline in transmission in Europe and the Americas was observed towards the end of 2022, with little understanding for the reasons underlying this change. We searched PubMed and MEDLINE from Jan 1, 2022, to March 31, 2024, with no language restrictions, using the search terms “monkeypox”, “mpox”, “sexual”, “behaviour”, “vaccine”, “vaccination”, and “transmission dynamics”. We found no multicountry reports on behavioural adaptation, naturally acquired immunity, or vaccination rates. At the country level, adaptations to sexual behaviour due to the outbreak have been independently described in Australia, Brazil, the Netherlands, the UK, and the USA. Modelling studies from Canada, Latin America, the Netherlands, the UK, and the USA have suggested that a combination of naturally acquired immunity and behavioural adaptation could explain decreases in transmission in these settings, due to insufficient vaccination coverage at the time of the decline.

**Added value of this study**

This retrospective, multiregional study provided insights into the extent and duration of behavioural adaptations among affected communities during the first year of the global mpox outbreak. Including more than 16 000 participants across 23 affected countries in Europe and the Americas, we characterised the widespread and dynamic nature of behavioural adaptations across contexts, including reductions in the number of sexual partners; avoidance of group sex, sexualised drug use (chemsex), and sex-on-premises venues; and engagement in open conversations about mpox with sexual partners. We identified regional variations in vaccination rates and the duration of behavioural adaptation, highlighting the complex interplay between individual risk perception, naturally acquired immunity, and vaccination strategies in shaping transmission dynamics.

**Implications of all the available evidence**

Behavioural adaptation and naturally acquired immunity had a primary role in the decline in transmission of MPXV clade IIb observed in Europe and the Americas at the end of 2022, particularly in settings with limited access to vaccination. During outbreaks, early access to vaccination for communities disproportionately affected by mpox is crucial to reduce harms attributed to illness and to the psychosocial burden of sustained behavioural adaptations. Risk-communication and community-engagement strategies are crucial to support affected communities in adopting protective measures, including making temporary and voluntary adaptations to sexual behaviour, as needed. These insights have implications for ongoing outbreak responses in sub-Saharan Africa, emphasising the urgent need to ensure equitable access to testing and vaccination and to implement robust risk-communication and community-engagement strategies.

Early epidemiological investigations found that MPXV clade IIb was transmitted in these newly affected countries in highly interconnected sexual networks of gay men and other men who have sex with men.^5^ Other groups affected included transgender and gender diverse people and sex workers.^6^ Self-reported sexual behaviours among people with mpox included having new sexual partners, visiting sex-on-premises venues such as sex clubs or saunas, engaging in group sex or sexualised drug use (hereafter referred to as chemsex), and meeting anonymous partners via geosocial networking smartphone-based apps.^7^, ^8^ Guidance issued by WHO starting in May, 2022, included extensive risk-communication and community-engagement strategies focused on providing affected communities with information about mpox and the sexual behaviours that were more likely to be associated with an increased risk of acquisition.^9^, ^10^ These strategies included the promotion of temporary adaptations to sexual behaviour as a protective measure, informed by engagement with representatives of the affected communities and civil society organisations.^9^, ^10^, ^11^ Pre-exposure and post-exposure vaccination prioritising the protection of the most affected communities was also recommended.^10^

After rapid epidemic growth between May and August, 2022, the number of new confirmed mpox cases declined in Europe and the Americas, with low-level transmission in these regions continuing thereafter while outbreaks increased in other regions.^3^ The reasons underpinning these rapid changes in transmission dynamics are unclear. Mathematical modelling data suggested that, in the UK, the decline in new diagnoses began too early to be attributed to the national vaccination strategy alone, suggesting that other factors—such as behavioural change and naturally derived immunity—might have initiated this decrease.^12^ In parallel, modelling from the USA, Canada, the Netherlands and Latin America suggested that adaptive behaviour change in affected sexual networks could drive a decrease in transmission of MPXV clade IIb.^13^, ^14^, ^15^

Although cases of mpox caused by MPXV clade IIb continue to be reported in all regions, the low transmission of MPXV clade IIb in Europe and the Americas was sustained throughout 2023 and early 2024.^16^ The main reasons for this sustained low transmission are still unclear, and the extent of adoption and duration of adaptations to sexual behaviour across these regions during the peak of the outbreak remains unknown. We aimed to characterise the presence and duration of adaptations to sexual behaviour related to mpox during the first year of the outbreak among affected communities in Europe and the Americas, in order to improve our understanding of transmission dynamics of MPXV clade IIb outside of west and central Africa and support the evaluation of risk-communication and community-engagement strategies used during the outbreak.

## Methods

### Study design and participants

This retrospective study used a cross-sectional online survey advertised through four apps (Grindr, Scruff, Jack'd, and Hornet) in 23 affected countries in Europe (Belgium, France, Germany, Ireland, Italy, the Netherlands, Poland, Portugal, Serbia, Spain, Switzerland, and the UK) and the Americas (Argentina, Brazil, Canada, Chile, Colombia, Costa Rica, Ecuador, Guatemala, Mexico, Peru, and the USA). Together, these countries accounted for more than 95% of all mpox cases reported during the outbreak at the time of the study.^3^ Inclusion criteria were agreeing to participate in the survey, being 18 years or older, and identifying as a gay man, a bisexual man, a man who has sex with men, as transgender, or as non-binary. The survey was live between May 19 and May 31, 2023. Participation in the study was voluntary; users were invited to participate through an advertisement for recruitment, which appeared as a direct message delivered to the user's inbox within the app or as a pop-up displayed when opening the app. This advertisement consisted of an invitation to participate in a survey about community experiences of the mpox outbreak and featured a link to the survey's landing page, which included information about the study's aim and procedures. After completing, participants were presented with sexual health information and messages and resources regarding mpox prevention.

Ethics approval was obtained from the WHO Ethics Research Committee (CERC.0194). The survey was anonymous and did not collect any identifiable information about participants or their devices, including IP addresses. Data on the participants' app use were not collected or available to the research team.

### Data collection and variables

Data were collected using an online questionnaire developed for the study, which was hosted on LimeSurvey. The questionnaire was developed in English, peer-validated by social scientists external to the core research team, and piloted with representatives of communities affected by mpox. The piloted version included a maximum of 32 multiple-choice and open-field questions and took an average of 4 min to complete. Participants could not skip questions unless they were not relevant based on answers previously provided. The final questionnaire (appendix p 1) was available in nine languages: Dutch, English, French, German, Italian, Polish, Portuguese, Serbian, and Spanish. All translations were validated by native speakers and community representatives to ensure that they were accurate and culturally appropriate. All data were stored on the platform servers at the WHO headquarters in Geneva, Switzerland. Aggregated data on the number of advertisement impressions and survey link clicks were provided by the apps.

The survey collected two primary outcome (dependent) variables: having made adaptations (made changes to sexual behaviour due to mpox concerns between May and December 2022, yes or no) and their continuation or reversal (still making changes to sexual behaviour in May 2023, yes or no). The period between May and December, 2022, was chosen because it corresponded with transmission peaks in the targeted countries.^3^ Secondary outcome variables were the presence of five specific adaptations to sexual behaviour (reduction in the number of sexual partners, avoidance of group sex, avoidance of sex-on-premises venues, avoidance of chemsex, and engagement in open conversations about mpox with sexual partners), and the specific duration of each of these adaptations (<1 month, 1 month to <2 months, 2 to <4 months, 4 to <12 months, and continuing adaptation).

Other variables collected included concerns about mpox (being concerned or worried about having mpox after finding out about the outbreak, yes or no), reasons for reversing adaptations to sexual behaviour (decrease in transmission, mpox vaccination, mpox diagnosis, and behavioural fatigue), willingness to adapt sexual behaviour if mpox transmission increased at a later time (yes or no), mpox diagnosis (yes or no; defined as having a diagnosis of mpox confirmed by a laboratory test, or as having symptoms compatible with mpox, since May, 2022), mpox vaccination with modified vaccinia virus Bavarian Nordic (two doses, one dose, or no vaccination), and access to vaccination among those unvaccinated (inability to access the vaccine, yes or no). For a full list of variables collected, see the study questionnaire (appendix pp 1–5).

We collected data on the following participant characteristics: age group, gender identity, sexual orientation, country of residence, history of sex work (currently doing sex work, have done sex work in the past but not currently, and have never done sex work), and HIV status (living with HIV, HIV negative, unknown HIV status, or prefer not to say; the unknown status and undisclosed status categories were combined for data analysis).

### Statistical analysis

Survey data were cleaned, managed, and analysed using Stata version 15.0. We calculated the click-through rate, a measure of engagement with advertisements in the apps, as the proportion of the number of clicks on the survey link to the number of advertisement impressions. We analysed only completed surveys (defined as the participant reaching the closing message that was displayed on the last page) from participants who met the study inclusion criteria. For analyses, participants were grouped into regions according to their country of residence: western Europe (Belgium, France, Germany, Ireland, Italy, the Netherlands, Portugal, Spain, Switzerland, and the UK), northern America (USA and Canada), Latin America (Argentina, Brazil, Chile, Colombia, Costa Rica, Ecuador, Guatemala, Mexico, and Peru), and eastern Europe and the western Balkans (Poland and Serbia). We summarised all variables using frequency tables and proportions. Proportions were compared using χ^2^ tests. For three of the specific adaptations to sexual behaviour (avoidance of group sex, avoidance of sex-on-premises venues, and avoidance of chemsex), proportions were calculated only among those who reported having engaged in these activities.

We estimated the association of each primary outcome with independent variables (age group, sexual orientation, gender identity, HIV status, sex work, and region) using generalised linear models with Poisson distribution and logistic link function with robust variances to estimate crude prevalence ratios (PRs) and prevalence ratios adjusted for confounding from the other covariates or predictors (aPRs). To estimate associations with the continuation or reversal of adaptations, we excluded participants who did not adapt their sexual behaviour and included mpox diagnosis and vaccination as additional independent variables. In bivariate analyses, the variable termed concerns about mpox was strongly associated with behavioural adaptation and with several independent variables. This variable was not included in final regression models because it was found to be a mediator in the causal pathway, leading to overadjustment.

### Role of the funding source

This study was funded, planned, and conducted by WHO.

## Results

The survey advertisements generated more than 2 million impressions, with a click-through rate ranging between 1·4% and 18·1% depending on the app and country. 24 306 people initiated the survey, with 17 428 (71·7%) completing it. People older than 25 years or those who identified as gay cisgender men were more likely to complete the survey (data not shown). Of 17 428 individuals who completed the survey, 16 875 (96·8%) met the eligibility criteria and were included in the study (figure). The median completion time was 4 min (IQR 2–4). Most of the 16 875 participants were cisgender men (16 081 [95·3%]) and identified as gay (13 560 [80·4%]) or bisexual (2784 [16·5%]; table 1). The majority of the participants were from western Europe (8097 [47·9%]) followed by Latin America (6166 [36·5%]), with smaller proportions from northern America (1994 [11·8%]), from eastern Europe and the western Balkans (511 [3·0%]), or not providing a country of residence (107 [0·6%]).

**Figure 1:**
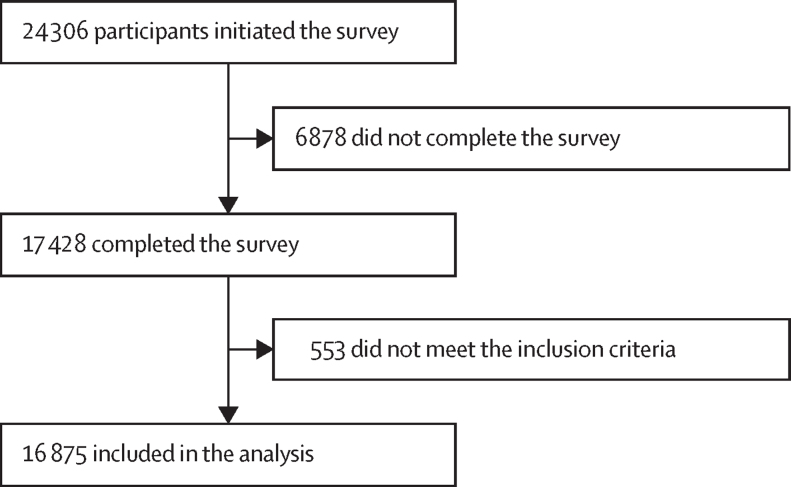
Study profile

**Table 1:** Characteristics of study participants

	**Number of participants (N=16 875)**
**Age group**	
18–25 years	2366 (14·0%)
26–34 years	4214 (25·0%)
35–44 years	4688 (27·8%)
45–54 years	3402 (20·2%)
≥55 years	2205 (13·1%)
**Gender identity**	
Cisgender man	16 081 (95·3%)
Transgender man	284 (1·7%)
Transgender woman	89 (0·5%)
Non-binary	265 (1·6%)
Other	156 (0·9%)
**Sexual orientation**	
Gay	13 560 (80·4%)
Bisexual	2784 (16·5%)
Heterosexual	202 (1·2%)
Other	329 (2·0%)
**Sex work**	
Never	14 636 (86·7%)
Currently	740 (4·4%)
In the past	1499 (8·9%)
**Region**	
Western Europe	8097 (47·9%)
Latin America	6166 (36·5%)
Northern America	1994 (11·8%)
Eastern Europe and the western Balkans	511 (3·0%)
Country of residence not provided	107 (0·6%)
**HIV status**	
Living with HIV	2974 (17·6%)
HIV negative	11 708 (69·4%)
Don't know or prefer not to say	2193 (13·0%)

Data are n (%). Percentages might not total 100 owing to rounding.

Table 2 shows the total and regional data for mpox diagnosis, vaccination, and behavioural adaptations. Of 16 875 participants, 1086 (6·4%) reported having mpox during the outbreak; prevalence was significantly lower among participants in eastern Europe and the western Balkans than among those in other regions (p<0·0001). Vaccination with at least one dose was reported by 4987 (29·6%) participants; 3502 (20·8%) reported two doses. Vaccination was rare in Latin America and eastern Europe and the western Balkans and significantly lower than in western Europe and northern America (p<0·0001). Among 11 888 unvaccinated participants, lack of access to vaccination was reported by 6953 (58·5%) and highest in Latin America (4528 [78·2%] of 5789 participants; p<0·0001). Participants who had concerns about mpox (9884 [58·6%] of 16 875) were more likely to have received at least one dose of the vaccine (3344 [33·7%] of 9884) than those who did not report having concerns (1653 [23·6%] of 6991; p<0·0001).

**Table 2:** Mpox diagnosis, vaccination, and behavioural adaption by region

		**Western Europe (N=8097)**	**Latin America (N=6166)**	**Northern America (N=1994)**	**Eastern Europe and the western Balkans (N=511)**	Total*(N=16 875)
**Mpox diagnosis**						
Yes		512 (6·3%)	386 (6·3%)	164 (8·2%)	17 (3·3%)	1086 (6·4%)
	Laboratory-confirmed	408 (5·0%)	277 (4·5%)	119 (6·0%)	6 (1·2%)	817 (4·8%)
	Compatible symptoms without laboratory confirmation	104 (1·3%)	109 (1·8%)	45 (2·3%)	11 (2·2%)	269 (1·6%)
No		7585 (93·7%)	5780 (93·7%)	1830 (91·8%)	494 (96·7%)	15 789 (93·6%)
**Mpox vaccination**						
Yes, two doses		2246 (27·7%)	215 (3·5%)	1023 (51·3%)	8 (1·6%)	3502 (20·8%)
Yes, one dose		1137 (14·0%)	162 (2·6%)	173 (8·7%)	7 (1·4%)	1485 (8·8%)
No		4714 (58·2%)	5789 (93·9%)	798 (40·0%)	496 (97·1%)	11 888 (70·4%)
**Lack of access to vaccination**†						
Yes		1885 (40·0%)	4528 (78·2%)	248 (31·1%)	247 (49·8%)	6953 (58·5%)
No		2829 (60·0%)	1261 (21·8%)	550 (68·9%)	249 (50·2%)	4935 (41·5%)
**Concerns about mpox**						
Yes		4526 (55·9%)	3920 (63·6%)	1254 (62·9%)	144 (28·2%)	9884 (58·6%)
No		3571 (44·1%)	2246 (36·4%)	740 (37·1%)	367 (71·8%)	6991 (41·4%)
**Adapted sexual behaviour**						
Yes		4035 (49·8%)	3178 (51·5%)	1229 (61·6%)	102 (20·0%)	8583 (50·9%)
No		4062 (50·2%)	2988 (48·5%)	765 (38·4%)	409 (80·0%)	8292 (49·1%)
**Duration of adaptations**‡						
Continued adaptations (up to May, 2023)		932 (23·1%)	1792 (56·4%)	266 (21·6%)	43 (42·2%)	3045 (35·5%)
Reversed adaptations (by May, 2023)		3103 (76·9%)	1386 (43·6%)	963 (78·4%)	59 (57·8%)	5538 (64·5%)
**Willingness to adapt during a future outbreak**						
Yes		6273 (77·5%)	5048 (81·9%)	1695 (85·0%)	381 (74·6%)	13 467 (79·8%)
No		1824 (22·5%)	1118 (18·1%)	299 (15·0%)	130 (25·4%)	3408 (20·2%)

Data are n (%). Percentages might not total 100 owing to rounding.

* Totals include 107 participants who did not provide their country of residence.

† Includes only unvaccinated participants (n=11 888).

‡ Includes only participants who adapted their sexual behaviour because of mpox between May and December, 2022 (n=8583).

Among the 16 875 study participants, 8583 (50·9%) reported adapting their sexual behaviour because of the mpox outbreak between May and December, 2022 (table 2). Among those who changed their behaviour, 7998 (93·2%) of 8583 reduced their number of sexual partners, 3815 (88·4%) of 4318 avoided group sex, 3534 (84·6%) of 4178 avoided sex-on-premises venues, 861 (53·6%) of 1607 avoided chemsex, and 4783 (55·7%) of 8583 talked openly about mpox with sexual partners (table 3). Behavioural adaptation was reported in all regions (table 2), but was significantly lower in eastern Europe and the western Balkans than in other regions (p<0·0001). For country-level data on mpox diagnosis, mpox vaccination, behavioural adaptation, and continuation of adaptations, see appendix p 6.

**Table 3:** Frequency and duration of specific behavioural adaptations due to mpox between May and December, 2022

	**Reducing number of sexual partners (N=8583)**	Avoiding group sex*(N=4318)	Avoiding sex-on-premises venues*(N=4178)	Avoiding chemsex*(N=1607)	**Having open conversations about mpox (N=8583)**
**Adapted sexual behaviour**					
Yes	7998 (93·2%)	3815 (88·4%)	3534 (84·6%)	861 (53·6%)	4783 (55·7%)
No	585 (6·8%)	503 (11·6%)	644 (15·4%)	746 (46·4%)	3800 (44·3%)
**Duration of adaptations (as of May, 2023)**†					
<1 month	392 (4·9%)	162 (4·2%)	128 (3·6%)	79 (9·2%)	630 (12·9%)
1 to <2 months	815 (10·2%)	311 (8·2%)	261 (7·4%)	62 (7·2%)	550 (11·5%)
2 to <4 months	1837 (23·0%)	760 (19·9%)	722 (20·4%)	109 (12·7%)	1037 (21·7%)
4 to <12 months	2151 (26·9%)	1166 (30·6%)	1220 (34·5%)	195 (22·7%)	988 (20·7%)
Continued adaptations	2803 (35·0%)	1416 (37·1%)	1203 (34·0%)	416 (48·3%)	1578 (33·0%)

Data are n (%). Percentages might not total 100 owing to rounding.

* Calculated only for participants who reported usually engaging in group sex, attending sex-on-premises venues, or engaging in chemsex, as applicable.

† Calculated only for participants who reported adapting their sexual behaviour.

In regression analyses (table 4), we found an association between having concerns about mpox and adapting sexual behaviour: participants who reported having concerns were more than twice as likely to report adaptations than those without concerns (PR 2·43 [95% CI 2·34–2·53]). In the adjusted model, participants from Latin America (aPR 1·07 [1·04–1·11]) or northern America (1·24 [1·19–1·29]) were more likely to have adapted their sexual behaviour than those from western Europe, and non-binary participants (1·16 [1·03–1·29]) were more likely to have made adaptations than those who identified as cisgender men. Conversely, participants aged 18–25, 26–34, and 55 years or older, those identifying their sexual orientation as heterosexual or other, those doing sex work currently or in the past, those residing in eastern Europe or the western Balkans, and those with unknown HIV status were less likely to have adapted their sexual behaviour than their respective counterparts (table 4).

**Table 4:** Adaptation to sexual behaviour due to mpox between May and December, 2022, by age group, gender identity, sexual orientation, sex work, region, HIV status, and concerns about mpox (N=16 875)

	**Adapted sexual behaviour**	**Did not adapt sexual behaviour**	**PR (95% CI)**	**aPR (95% CI)**
**Age group**				
18–25 years	910/2366 (38·5%)	1456/2366 (61·5%)	0·69 (0·65–0·73)	0·73 (0·69–0·78)*
26–34 years	2188/4214 (51·9%)	2026/4214 (48·1%)	0·93 (0·90–0·97)	0·94 (0·91–0·98)*
35–44 years	2615/4688 (55·8%)	2073/4688 (44·2%)	Ref	Ref
45–54 years	1835/3402 (53·9%)	1567/3402 (46·1%)	0·97 (0·93–1·01)	0·96 (0·92–1·00)
≥55 years	1035/2205 (46·9%)	1170/2205 (53·1%)	0·84 (0·80–0·89)	0·82 (0·77–0·85)*
**Gender identity**				
Cisgender man	8198/16 081 (51·0%)	7883/16 081 (49·0%)	Ref	Ref
Transgender man	136/284 (47·9%)	148/284 (52·1%)	0·94 (0·83–1·06)	1·04 (0·92–1·18)
Transgender woman	40/89 (44·9%)	49/89 (55·1%)	0·88 (0·70–1·11)	1·09 (0·86–1·39)
Non-binary	145/265 (54·7%)	120/265 (45·3%)	1·07 (0·96–1·20)	1·16 (1·03–1·29)*
Other	64/156 (41·0%)	92/156 (59·0%)	0·80 (0·67–0·97)	0·90 (0·74–1·08)
**Sexual orientation**				
Gay	7066/13 560 (52·1%)	6494/13 560 (47·9%)	Ref	Ref
Bisexual	1312/2784 (47·1%)	1472/2784 (52·9%)	0·90 (0·87–0·94)	0·95 (0·91–0·99)*
Heterosexual	78/202 (38·6%)	124/202 (61·4%)	0·74 (0·62–0·88)	0·80 (0·67–0·95)*
Other	127/329 (38·6%)	202/329 (61·4%)	0·74 (0·65–0·85)	0·77 (0·67–0·89)*
**Sex work**				
Never	7557/14 636 (51·6%)	7079/14 636 (48·4%)	Ref	Ref
Currently	310/740 (41·9%)	430/740 (58·1%)	0·81 (0·74–0·88)	0·85 (0·78–0·93)*
In the past	716/1499 (47·8%)	783/1499 (52·2%)	0·93 (0·88–0·98)	0·93 (0·88–0·98)*
**Region**				
Western Europe	4035/8097 (49·8%)	4062/8097 (50·2%)	Ref	Ref
Latin America	3178/6166 (51·5%)	2988/6166 (48·5%)	1·03 (1·00–1·07)	1·07 (1·04–1·11)*
Northern America	1229/1994 (61·6%)	765/1994 (38·4%)	1·24 (1·19–1·29)	1·24 (1·19–1·29)*
Eastern Europe and the western Balkans	102/511 (20·0%)	409/511 (80·0%)	0·40 (0·33–0·47)	0·41 (0·35–0·50)*
**HIV status**				
Living with HIV	1585/2974 (53·3%)	1389/2974 (46·7%)	1·02 (0·98–1·06)	0·99 (0·95–1·03)
HIV negative	6100/11 708 (52·1%)	5608/11 708 (47·9%)	Ref	Ref
Don't know or prefer not to say	898/2193 (41·0%)	1295/2193 (59·0%)	0·79 (0·75–0·83)	0·85 (0·81–0·90)*
**Concerns about mpox**				
Yes	6650/9884 (67·3%)	3234/9884 (32·7%)	2·43 (2·34–2·53)	..
No	1933/6991 (27·6%)	5058/6991 (72·4%)	Ref	..

Data are n/N (%) unless otherwise indicated. Percentages might not total 100 owing to rounding. PR=prevalence ratio. aPR=adjusted prevalence ratio.

* Indicates significant differences from the reference value.

Among the 8583 participants who made adaptations, 3045 (35·5%) said they had continued to adapt their sexual behaviour due to the outbreak up to May, 2023, whereas 5538 (64·5%) made adaptations between May and December, 2022, but reversed them by May, 2023 (table 2). For all adaptations, more than half of the participants who reported making the adaptation did so for at least 4 months (table 3). Reasons given by the 5538 participants who reversed their adaptations by May, 2023 were a decline in mpox transmission (3451 [62·3%]), having had mpox vaccination (2481 [44·8%]), behavioural fatigue (1098 [19·8%]), and having had mpox (362 [6·5%]).

Participants who reported having concerns about mpox and who made adaptations to their sexual behaviour were less likely to continue with these adaptations by May, 2023, than those who made adaptations but did not report concerns (PR 0·75 [95% CI 0·70–0·80]; table 5). In the adjusted model, participants who reported being vaccinated were less likely to continue adapting their sexual behaviour than those unvaccinated. Similarly, participants who had mpox were less likely to continue adaptations than those who did not have mpox. People in Latin America, northern America, and eastern Europe and the western Balkans were significantly more likely to continue with adaptations than those in western Europe. Most participants (13 467 [79·8%] of 16 875) reported willingness to adapt their sexual behaviour if mpox transmission rapidly increased in their community; this proportion was more than 70% across all regions (table 2).

**Table 5:** Continuation and reversal of adaptations to sexual behaviour due to mpox in May, 2023, among those who changed their behaviour (n=8583*)

	**Continued adaptations (up to May, 2023)**	**Reversed adaptations (by May, 2023)**	**PR (95% CI)**	**aPR (95% CI)**
**Age group**				
18–25 years	462/910 (50·8%)	448/910 (49·2%)	1·58 (1·45–1·72)	1·02 (0·94–1·10)
26–34 years	832/2188 (38·0%)	1356/2188 (62·0%)	1·18 (1·09–1·28)	0·97 (0·91–1·05)
35–44 years	842/2615 (32·2%)	1773/2615 (67·8%)	Ref	Ref
45–54 years	578/1835 (31·5%)	1257/1835 (68·5%)	0·98 (0·90–1·07)	1·04 (0·96–1·12)
≥55 years	331/1035 (32·0%)	704/1035 (68·0%)	0·99 (0·89–1·10)	1·12 (1·02–1·24)†
**Gender identity**				
Cisgender man	2868/8198 (35·0%)	5330/8198 (65·0%)	Ref	Ref
Transgender man	55/136 (40·4%)	81/136 (59·6%)	1·16 (0·94–1·42)	1·20 (0·89–1·62)
Transgender woman	23/40 (57·5%)	17/40 (42·5%)	1·64 (1·26–2·15)	1·11 (0·89–1·38)
Non-binary	66/145 (45·5%)	79/145 (54·5%)	1·30 (1·19–1·56)	1·16 (0·98–1·37)
Other	33/64 (51·6%)	31/64 (48·4%)	1·47 (1·16–1·87)	1·22 (1·01–1·48)†
**Sexual orientation**				
Gay	2319/7066 (32·8%)	4747/7066 (67·2%)	Ref	Ref
Bisexual	627/1312 (47·8%)	685/1312 (52·2%)	1·46 (1·36–1·55)	1·14 (1·07–1·21)
Heterosexual	49/78 (62·8%)	29/78 (37·2%)	1·91 (1·61–2·28)	1·22 (0·99–1·51)
Other	50/127 (39·4%)	77/127 (60·6%)	1·20 (0·96–1·49)	0·94 (0·77–1·15)
**Sex work**				
Never	2626/7557 (34·8%)	4931/7557 (65·3%)	Ref	Ref
Currently	139/310 (44·8%)	171/310 (55·2%)	1·29 (1·13–1·47)	1·07 (0·94–1·22)
In the past	280/716 (39·1%)	436/716 (60·9%)	1·13 (1·02–1·24)	1·01 (0·93–1·11)
**Region**				
Western Europe	932/4035 (23·1%)	3103/4035 (76·9%)	Ref	Ref
Latin America	1792/3178 (56·4%)	1386/3178 (43·6%)	2·44 (2·28–2·60)	1·67 (1·56–1·78)†
Northern America	266/1229 (21·6%)	963/1229 (78·4%)	0·94 (0·83–1·06)	1·28 (1·14–1·43)†
Eastern Europe and the western Balkans	43/102 (42·2%)	59/102 (57·8%)	1·83 (1·45–2·32)	1·29 (1·03–1·62)†
**HIV status**				
Living with HIV	520/1585 (32·8%)	1065/1585 (67·2%)	0·96 (0·89–1·04)	0·92 (0·86–0·99)†
HIV negative	2082/6100 (34·1%)	4018/6100 (65·9%)	Ref	Ref
Don't know or prefer not to say	443/898 (49·3%)	455/898 (50·7%)	1·45 (1·34–1·55)	1·06 (0·99–1·14)
**Mpox diagnosis**				
Yes‡	101/668 (15·1%)	567/668 (84·9%)	0·50 (0·41–0·60)	0·37 (0·30–0·44)†
No	2944/7915 (37·2%)	4971/7915 (62·8%)	Ref	Ref
**Mpox vaccination**				
Yes, two doses	235/2322 (10·1%)	2087/2322 (89·9%)	0·21 (0·18–0·23)	0·25 (0·21–0·28)†
Yes, one dose	142/830 (17·1%)	688/830 (82·9%)	0·35 (0·30–0·41)	0·43 (0·37–0·51)†
No	2668/5431 (49·1%)	2763/5431 (50·9%)	Ref	Ref
**Concerns about mpox**				
Yes	2193/6650 (33·0%)	4457/6650 (67·0%)	0·75 (0·70–0·80)	..
No	852/1933 (44·1%)	1081/1933 (55·9%)	Ref	..

Data are n/N (%) unless otherwise indicated. Percentages might not total 100 owing to rounding. PR=prevalence ratio. aPR=adjusted prevalence ratio.

* Includes only those who adapted their sexual behaviour because of mpox between May and December, 2022.

† Indicates significant differences from the reference value.

‡ Includes those with either a laboratory diagnosis or with compatible symptoms during the outbreak.

## Discussion

This retrospective multiregional study found that adaptations to sexual behaviour were widespread, dynamic, and based on evolving individual risk perceptions among affected communities during the first year of the global outbreak of MPXV clade IIb. A moderate degree of lasting behavioural adaptation persisted across all included countries after 1 year, particularly among participants who were still considered to be susceptible to mpox. Given stark vaccine inequity but similar reductions in transmission rates between regions during the first year of the global response, our study supports the hypothesis that the sudden decline in MPXV clade IIb transmission seen at the end of 2022 was primarily due to a combination of behavioural adaptation and naturally acquired immunity.

More than half of the participants adapted their sexual behaviour by reducing their number of partners, avoiding group sex, or avoiding sex-on-premises venues, closely mirroring findings from other single-country studies conducted in Australia, Brazil, the Netherlands, the UK, and the USA.^17^, ^18^, ^19^, ^20^, ^21^ Our findings indicate that behavioural adaptations highlighted in early risk-communication messaging were recognised by affected communities as feasible to mitigate the risk of acquiring mpox, possibly due to involvement of these communities in developing this messaging. Participants aged 34 years and younger and those aged 55 years and older were less likely to make behavioural adaptations than participants aged 35–44 years. This finding could be explained by a lower number of sexual partners in these age groups and a reduced perceived risk of mpox acquisition.^22^ Non-binary participants could have been more likely to adapt their behaviours than cisgender men due to being more connected to sexual health messaging, whereas increased adaptation in the Americas compared with Europe could be attributed to the delayed onset of the outbreak in this region—allowing more time for the dissemination and adoption of public health advice—and to greater concern due to reduced vaccine accessibility.

We also found that more than half of the participants who adapted their behaviours did so for 4 months or longer, with more than a third of those who made adaptations continuing them 1 year after the outbreak started. We found a strong dose–response association between being vaccinated and reversing adaptations, and a strong association between having mpox and reversing the changes made. These findings suggest that adaptations were dynamic during the outbreak due to changes in risk perception, as further evidenced by a decrease in mpox transmission being reported as the primary reason to reverse the adaptations made. Having concerns about acquiring mpox was associated with a higher likelihood of behavioural adaptation, vaccination, and reversal of adaptations. Overall, these findings align with those of other studies in which the perceived susceptibility to and severity of mpox, and the changes of these perceptions in time, were described as motivators for risk-reduction strategies.^18^, ^19^, ^23^ The reversal of adaptations among those who had concerns could be explained by higher vaccination rates in this group and less concern over time: studies in the USA found that being vaccinated for mpox was associated with more sexual activity during the outbreak than being unvaccinated, and that having fewer concerns about mpox over time was associated with reduced behavioural adaptation.^24^, ^25^ Notably, participants aged 55 years and older were less likely to make adaptations but more likely to continue them over time, which could be due to previous health experiences or perceived protection by previous smallpox vaccination.

This study shows stark regional contrasts in mpox vaccination rates by May, 2023, strongly suggesting that the rapid decrease in cases reported by December, 2022, was not a result of vaccine-acquired immunity. This suggestion is best evidenced by a two-dose vaccination rate of 3·5% among participants in Latin America in May, 2023, at a time of sustained low-level transmission, and by a modelling study that found that the decline in transmission in Latin America occurred before the roll-out of vaccination strategies.^3^, ^15^ Taken together, these findings support a growing body of evidence indicating that the rapid decline in MPXV clade IIb transmission in Europe and the Americas resulted from a combination of behavioural adaptation and naturally acquired immunity.^12^, ^15^, ^21^, ^26^, ^27^ Notably, a modelling study suggested that vaccination expedited the resolution of the outbreak in the USA.^28^ Knowledge gaps remain on the durability of vaccine-induced and naturally induced immune responses.

This study consistently identified an mpox prevalence of greater than 6·0% among study participants in all regions except eastern Europe and the western Balkans. Mpox modelling simulations using data from the UK found that epidemics can reach the threshold of naturally derived herd immunity and subsequently decrease even with less than 1% prevalence among sexually active men who have sex with men regardless of interventions or behavioural changes.^27^ This finding is explained by the heavy-tailed distribution of sexual partners within these networks, whereby a small proportion of the network accounts for a relatively high number of sexual partnerships.^26^ Although our survey findings might overestimate the prevalence of mpox generally owing to participation bias, we identified evidence of some degree of naturally acquired immunity within these networks across all regions. In combination with unequal and insufficient vaccination coverage, our findings suggest that naturally derived immunity had a primary role in reducing transmission, particularly in areas with limited or no access to mpox vaccines.

The study characterises the extent and persistence of behavioural adaptation among affected communities. A study modelling the effect of changes in sexual behaviour among men who have sex with men identified that a 40% reduction in one-time partnerships could delay MPXV clade IIb transmission and reduce the proportion of people affected by 20–31%.^13^ Moreover, a modelling study of transmission dynamics in Washington, DC, USA, found that the initial decline in cases was due to the magnitude of behavioural adaptation reported by affected communities.^28^ This reason was also proposed by transmission dynamic modelling studies in the UK and in Latin America.^15^, ^29^ Owing to the magnitude of adoption and duration of behavioural adaptations found in our study, we propose that behaviour change had a primary role in reducing transmission, although our study was not designed to assess whether this role superseded that of naturally acquired immunity. Future mathematical modelling and seroprevalence studies might be able to address this knowledge gap using data from our study as parameters to estimate transmission dynamics.

Some key regional variations were identified in this study. First, Latin America had the longest duration of behavioural adaptation, which could be due to significantly lower access to vaccination in this region than in Europe and northern America. These findings suggest that the risk of mpox resurgence could be high in Latin America.^15^, ^30^ Second, concerns regarding mpox and behavioural adaptation due to the outbreak were lower in eastern Europe and the western Balkans than in any other region. The reduced risk perception and behavioural adaptation observed align with a smaller outbreak in this region, as evidenced by the lower reported history of mpox in the survey and by global surveillance.^3^ We note that individuals who adapted their behaviour in this region tended to do so for longer periods, possibly because of limited access to vaccination among those with perceived risk.

The implications of this study for risk-communication and community-engagement strategies include a need to provide focused support to communities during periods of high mpox transmission. Where a lack of information and stigma continue to limit diagnosis and access to care, outcomes can be devastating, as shown in the outbreak due to clade IIb MPXV in South Africa. Moreover, the ongoing outbreak of clade Ib MPXV in east-central Africa involves a high proportion of sex workers, for whom voluntary adaptation of their behaviour might be much more challenging.^31^ In these settings, providing focused support to commercial sex venues and communities of sex workers during periods of high transmission might be necessary to support behavioural adaptations and ensure access to vaccines as soon as they become available.

This study has several limitations. First, we used a non-random and convenience-based sample, which might not be representative of all members of affected communities in the study regions. Participation bias might also have led those who are more health-conscious or community-engaged to participate, potentially overestimating the degree of behavioural adaptation. Complete surveys were more frequent among those who were older than 25 years, gay, or men, suggesting some degree of self-selection. Furthermore, app users tend to have higher-risk sexual behaviour than non-users, which could reduce the generalisability of these findings to wider sexual networks who do not use these apps.^32^ Different app usage habits across regions could introduce a degree of selection bias. Finally, self-reported data can introduce recall bias and, to a lesser extent, social desirability bias, potentially influencing the accuracy of reported behaviour changes, mpox prevalence, and vaccination rates. Additional efforts within the social sciences are needed to characterise and understand in-depth and context-specific adaptations to sexual behaviour among affected communities.

This study provides comprehensive evidence on the magnitude of behavioural adaptation, naturally acquired immunity, and vaccination during the first year of the MPXV clade IIb outbreak, supporting our understanding of the decrease in transmission and informing ongoing and future outbreak responses. Our findings emphasise the importance of individual risk perception in adopting protective measures against mpox and underscore the need for robust risk-communication and community-engagement strategies in outbreaks that affect sexual networks in any context. These strategies are crucial to support individuals to dynamically assess their risks and take necessary preventive measures in the event of an outbreak. The high willingness among affected communities to adapt in case of a future mpox resurgence, and the high reach of the study's advertisement campaign, present clear opportunities for further risk-communication and community-engagement work in Europe and the Americas. Urgent actions are needed to ensure global equity in accessing vaccination and testing, particularly in countries in sub-Saharan Africa where transmission is increasing. Early access to mpox vaccination in the event of an outbreak is crucial to avoid harm to communities due to illness and to minimise the psychosocial costs of sustained behavioural adaptations, especially in contexts in which behavioural adaptation might be challenging.

### Contributors

### Data sharing

Individual-level underlying data can be shared if appropriate, subject to approval by the WHO Ethics Research Committee, via request to the corresponding author.

## Declaration of interests

We declare no competing interests. The authors affiliated with WHO are alone responsible for the views expressed in this publication and they do not necessarily represent the decisions or policies of WHO.
