# Supplementary material for: Temporary adaptations to sexual behaviour during the mpox outbreak in 23 countries in Europe and the Americas: findings from a retrospective cross-sectional online survey
Source: Lancet Infect Dis. 2024 Dec;24(12):1309–18. doi: 10.1016/S1473-3099(24)00531-0 (PMC11810863; doi:10.1016/S1473-3099(24)00531-0)
Supplement: Supplementary appendix [file mmc1.pdf]

THE LANCET
Infectious Diseases

Supplementary appendix

This appendix formed part of the original submission and has been peer reviewed. We post it as supplied by the authors.

Supplement to: Prochazka M, Vinti P, Hoxha A, et al. Temporary adaptations to sexual behaviour during the mpox outbreak in 23 countries in Europe and the Americas: findings from a retrospective cross-sectional online survey. *Lancet Infect Dis* 2024; published online Sept 18. https://doi.org/10.1016/S1473-3099(24)00531-0.

Supplementary Material 1 – Online questionnaire

For information page:
This survey is launched by the World Health Organization to identify how the mpox (monkeypox) outbreak changed the sexual lives of people who use this application. It is completely anonymous and will not be linked to any data from your app usage.
Your participation is completely optional and it will take up to 5 minutes of your time.

By agreeing to start this survey you are consenting to the use of your anonymised data for research purposes.

Part 1: Screening

1. **How did you find this survey?**
    - Grindr
    - Scruff
    - Jack'd
    - Hornet
    - Other

2. **How old are you?**
    - Less than 18 years old → *end survey*
    - 18-25 years old
    - 26-34 years old
    - 35-44 years old
    - 45-54 years old
    - 55 years old or more

3. **Do you identify as gay man, bisexual man, or a man who has sex with men?**
    - Yes
    - No

4. **Do you identify as trans, transgender, or non-binary?**
    - Yes → *continue to question 5*
    - No → *continue to question 5 if question 3 is Yes, end survey if question 3 is No*

Part 2: Demographics

5. **Which of the following best describes you?**
    - I am a man
    - I am a woman
    - I am non-binary
    - In another way

6. **Which of the following best describes you?**
    - I am gay or homosexual
    - I am bisexual
    - I am straight or heterosexual
    - In another way

7. **Which of the following best describes you?**
    - I do sex work
    - I have never done sex work
    - I do not do sex work currently, but have done sex work in the past

8. **What is your country of residence?**
    - List of countries (dropdown menu)

<u>Part 3: Mpox history</u>

9. **During 2022-23, were you ever diagnosed with mpox (monkeypox)?**
    - Yes, I was diagnosed with it → *continue to question* 10
    - No, I was not diagnosed but I think I had it → *continue to question 10*
    - No, I never had it → *jump to question 11*
    - I don't know → *jump to question 11*

10. **When did you have a confirmed or possible mpox diagnosis?**
    - Month/Year

11. **Have you had a monkeypox (mpox) vaccine?**
    - Yes, 1 dose → *continue to question 12*
    - Yes, 2 doses → *continue to question 12*
    - No → *jump to question 13*

12. **When did you receive your mpox vaccines?**
    - First dose
        - Month (number)
        - Year
    - Second dose (leave blank if not received)
        - Month (number)
        - Year

13. **How would you best describe your reason for not being vaccinated against mpox? (mark all that apply)**
    - I cannot access this vaccine
    - I do not need this vaccine
    - I do not want to receive this vaccine

<u>Part 4: Mpox concerns</u>

14. **After finding out about the outbreak, were you concerned or worried about having mpox?**
    - Yes → *continue to question 15*
    - No, I did not experience any concerns → *jump to question 17*

15. **What concerns did you have? (mark all that apply)**
    - Pain
    - Skin marks or scars
    - Shame or feeling bad about myself
    - Being discriminated or stigmatized by others
    - Having to self-isolate
    - Impact on my sexual life
    - Impact on my employment
    - Not being able to access health care
    - Possibility of dying
    - Other concerns → *show question 16*

16. **What other concerns did you have?**
    - Open field

<u>Part 5: Changes in sexual behaviour</u>

17. **Between May and December 2022, did you change your sexual behaviour due to mpox concerns, even if temporarily?**
    Changes in your sexual behaviour can include: reducing number of sexual partners, avoiding group sex, avoiding visiting sex clubs or saunas, avoiding using drugs in sexual contexts, or openly discussing mpox with your sexual partners.

    - Yes, I changed my sexual behaviour due to mpox → continue to question 18
    - No, I did not change my sexual behaviour due to mpox → jump to question 29

18. **Between May and December 2022, did you reduce the number of sexual partners you had at any time due to mpox concerns?**
    - Yes, I did → *continue to question 19*
    - No, I did not → *jump to question 20*

19. **For how long did you reduce the number of sexual partners you had at any time due to mpox concerns?**
    - A few days to 1 week
    - 1 week to less than a 1 month
    - 1 month to less than 2 months
    - 2 months to less than 4 months
    - 4 months or more
    - I am still reducing the number of sexual partners I have

20. **Between May and December 2022, did you avoid group sex at any time due to mpox concerns?**
    - Yes, I did → *continue to question 21*
    - No, I did not → *jump to question 22*
    - I do not usually practice group sex → *jump to question 22*

21. **For how long did you avoid group sex due to mpox concerns?**
    - A few days to 1 week
    - 1 week to less than a 1 month
    - 1 month to less than 2 months
    - 2 months to less than 4 months
    - 4 months or more
    - I am still avoiding group sex

22. **Between May and December 2022, did you avoid visiting sex clubs or saunas due to mpox concerns?**
    - Yes, I did → *jump to question 23*
    - No, I did not → *jump to question 24*
    - I do not usually visit sex clubs or saunas → *jump to question 24*

23. **For how long did you avoid visiting sex clubs or saunas due to mpox concerns?**
    - A few days to 1 week
    - 1 week to less than a 1 month
    - 1 month to less than 2 months
    - 2 months to less than 4 months

- 4 months or more
- I am still avoiding visiting sex clubs or saunas

24. Between May and December 2022, did you avoid using drugs in sexual contexts at any time due to mpox concerns?
- Yes, I did → *continue to question 25*
- No, I did not → *jump to question 26*
- I do not usually use drugs in sexual contexts → *jump to question 26*

25. For how long did you avoid using drugs in sexual contexts due to mpox concerns?
- A few days to 1 week
- 1 week to less than a 1 month
- 1 month to less than 2 months
- 2 months to less than 4 months
- 4 months or more
- I am still avoiding using drugs in sexual contexts

26. Between May and December 2022, did you openly discuss mpox with sexual partners at any time?
- Yes, I did → *continue to question 27*
- No, I did not → *jump to question 28*

27. For how long did you openly discuss mpox with sexual partners?
- A few days to 1 week
- 1 week to less than a 1 month
- 1 month to less than 2 months
- 2 months to less than 4 months
- 4 months or more
- I am still openly discussing the risk of mpox with sexual partners

28. Why did you stop any changes to your sexual behaviour? (mark all that apply)
- I got tired of changing my sexual behaviour
- I got my mpox vaccine
- I was diagnosed with mpox
- Transmission decreased or the outbreak ended
- I did not reverse any of the changes

29. If there was a rapid increase in mpox cases in your community, would you take any of the following actions? (mark all that apply)
- Reduce the number of sexual partners
- Avoid group sex
- Avoid visiting sex clubs or saunas
- Avoid using drugs or alcohol in sexual contexts
- Openly discuss mpox with sexual partners
- Get vaccinated for mpox
- I would not make any changes
- Other (open field)

Part 6: HIV questions

30. Do you know your HIV status?

People with HIV can live a long and healthy life if they are aware of their HIV status and receive effective treatment.

- I am HIV positive → *continue to question 31*
- I am HIV negative → *jump to question 32*
- I don't know my HIV status → *end survey*
- Prefer not to say → *end survey*

31. Are you on antiretroviral treatment?

People with HIV who take effective treatment achieve viral suppression (their viral load is undetectable), which means they cannot pass HIV to their partners.

- Yes, I am on antiretroviral treatment and my viral load is undetectable → *end survey*
- Yes, I am on antiretroviral treatment and my viral load is not undetectable → *end survey*
- No, I am not on antiretroviral treatment → *end survey*

32. Are you on pre-exposure prophylaxis (PrEP) for HIV?

PrEP is one of most effective ways to prevent HIV. If you are not on PrEP, you can discuss with your healthcare provider whether you may benefit from it.

- Yes, I am on PrEP → *end survey*
- No, I am not on PrEP → *end survey*
- I am not on PrEP but I am considering to start it soon → *end survey*

Closing message

Thank you for participating in this survey.

Please consider testing regularly for sexually-transmitted infections (STIs) including HIV if you don't know your status.

Mpox can spread during close contact, including sex. To protect yourself and others you can:

- *Stay informed about the risk of mpox in your community*
- *Have open conversations about mpox with close contacts and partners*
- *Avoid close contact with people with mpox while they recover*
- *Monitor closely for symptoms if you have been exposed*
- *Get tested if you have mpox symptoms such as a new rash or rectal pain*
- *Get vaccinated if this is available*
- *Keep taking steps to protect yourself and others, even after vaccination*

You can help combating misinformation by sharing only trustworthy and non-stigmatizing information from official sources.

Supplementary table 1. Country-level data on mpox diagnosis, mpox vaccination, behavioural adaptation and continuation of adaptations

Country (N)	Mpox diagnosis* % (n)	Mpox diagnosis (laboratory confirmed only) % (n)	Mpox vaccination (2 doses) % (n)	Mpox vaccination (only 1 dose) % (n)	Adapted sexual behaviour % (n)	Continued adaptations by May 2023 % (n/total**)
Argentina (624)	3.9 (24)	2.7 (17)	0.6 (4)	1.1 (7)	32.1 (200)	55.5 (111/200)
Belgium (136)	6.6 (9)	5.2 (7)	34.6 (47)	11.8 (16)	57.4 (78)	16.7 (13/78)
Brazil (3,043)	6.4 (194)	4.6 (139)	2.5 (77)	2.5 (76)	54.2 (1,648)	60.9 (1,004/1,648)
Canada (377)	4.5 (17)	4.0 (15)	45.6 (172)	19.4 (73)	57.6 (217)	20.7 (45/217)
Chile (181)	5.5 (10)	3.3 (6)	23.2 (42)	6.6 (12)	50.3 (91)	45.1 (41/91)
Colombia (135)	9.6 (13)	8.2 (11)	0 (0)	1.5 (2)	51.1 (69)	47.8 (33/69)
Costa Rica (188)	7.5 (14)	3.7 (7)	0.5 (1)	0.5 (1)	45.2 (85)	67.1 (57/85)
Ecuador (94)	7.5 (7)	3.2 (3)	2.1 (2)	3.2 (3)	42.6 (40)	57.5 (23/40)
France (1,279)	5.3 (68)	4.3 (55)	31.4 (402)	27.2 (348)	49.2 (629)	14.9 (94/629)
Germany (1,285)	6.6 (90)	5.0 (68)	35.1 (483)	14.3 (197)	47.3 (650)	16.5 (107/650)
Guatemala (75)	9.3 (7)	6.7 (5)	0.0 (0)	2.7 (2)	60.0 (45)	66.7 (30/45)
Ireland (59)	1.7 (1)	1.7 (1)	59.3 (35)	6.8 (4)	52.5 (31)	16.1 (5/31)
Italy (654)	4.3 (28)	3.7 (24)	27.5 (180)	8.4 (55)	37.6 (246)	24.8 (61/246)
Mexico (1,370)	6.4 (87)	4.7 (64)	2.0 (28)	2.2 (30)	54.2 (742)	51.2 (380/742)
The Netherlands (297)	6.7 (20)	4.0 (12)	30.3 (90)	19.9 (59)	43.1 (128)	16.4 (21/128)
Peru (416)	6.5 (27)	5.5 (23)	13.0 (54)	6.3 (26)	57.0 (237)	42.2 (100/237)
Poland (459)	3.1 (14)	1.1 (5)	1.5 (7)	1.3 (6)	17.9 (82)	42.7 (35/82)
Portugal (661)	4.2 (28)	3.5 (23)	13.8 (91)	6.4 (42)	54.2 (358)	42.5 (152/358)
Serbia (42)	0 (0)	0 (0)	0 (0)	2.4 (1)	40.5 (17)	41.2 (7/17)
Spain (2,200)	8.9 (196)	7.5 (164)	17.2 (379)	9.6 (212)	53.0 (1165)	29.5 (344/1,165)
Switzerland (189)	6.4 (12)	4.8 (9)	43.9 (83)	10.1 (19)	58.7 (111)	11.7 (13/111)
United Kingdom (1,186)	4.6 (55)	3.5 (42)	37.2 (441)	14.9 (177)	51.1 (606)	19.1 (116/606)
United States of America (1,613)	9.1 (146)	6.5 (104)	52.6 (849)	6.2 (100)	62.7 (1011)	21.9 (221/1,011)

*Includes both laboratory-confirmed and those with compatible symptoms but without laboratory confirmation
**Only includes those who adapted their sexual behaviour due to mpox
